# The Association Between Social Support, COVID-19 Exposure, and Medical Students' Mental Health

**DOI:** 10.3389/fpsyt.2021.555893

**Published:** 2021-05-24

**Authors:** Yi Yin, Xingjie Yang, Lan Gao, Suoyuan Zhang, Meng Qi, Ligang Zhang, Yunlong Tan, Jingxu Chen

**Affiliations:** ^1^Peking University HuiLongGuan Clinical Medical School, Beijing HuiLongGuan Hospital, Beijing, China; ^2^Beijing Suicide Research and Prevention Center, WHO Collaborating Center for Research and Training in Suicide Prevention, Beijing, China; ^3^Chengde Medical University, Chengde, China

**Keywords:** depression, anxiety, COVID-19, medical students, exposure, social support

## Abstract

**Background:** The coronavirus disease−2019 (COVID-19) pandemic has halted in-person medical education worldwide. Limited studies have reported on the mental health status of medical students during this public health emergency. This study aimed to explore the association of personal virus exposure, regional epidemic condition, and social support with medical students' depressive and anxiety symptoms during the COVID-19 outbreak in China.

**Methods:** In February 2020, 5,982 medical students (60.0% females, Mean_age_ = 21.7 years, Median_age_ = 22 years) completed an online survey consisting of demographics, personal virus exposure, the Patient Health Questionnaire, the Generalized Anxiety Disorder Scale, and the Social Support Rating Scale.

**Results:** The prevalence rates of mild to severe depressive symptoms and anxiety symptoms were 35.2 and 22.8%, respectively. Multivariate linear regression showed that students with low- or medium-level social support had a higher risk of experiencing depressive or anxiety symptoms than those with high-level social support. COVID-19 exposure was positively associated with mild to severe depressive or anxiety symptoms. Respondents living in provinces with 500–1,000 confirmed COVID-19 cases had an increased risk of experiencing mild to severe depressive symptoms compared with those living in provinces with <100 cases. Other related factors were gender and years of training.

**Conclusions:** Some medical students suffered from a poor psychological status during the COVID-19 outbreak. Low social support was a stronger factor related to poor mental status compared with COVID-19 exposure or the provincial epidemic condition. Thus, we suggest that colleges or universities provide social support and mental health screening.

## Introduction

Newly emerging infectious diseases are black swan incidents that are challenging for global healthcare systems. National capacities for coping with public health emergencies rely heavily on healthcare human resource preparedness. It is questionable whether mass news coverage, potential exposure to coronavirus disease−2019 (COVID-19), and possible suspended education (1, 2) can impact medical students. Medical students have multiple identities, such as future healthcare workers, young adults, and common citizens. Their emotions and psychological suffering during this outbreak may provide important information toward preparing human resources for further health emergencies.

Meta-analyses showed that, on normal days, the prevalence rates of depression and anxiety among all medical students were approximately 27% (3–5) and 30% (6), respectively. The risk factors were being female, receiving 1st year undergraduate or postgraduate education, and poor psychological support (5, 7, 8).

The COVID-19 pandemic has influenced the mental health status of college or university students. A meta-analysis showed that approximately one-third of college or university students had depressive or anxiety symptoms (9). Different testing procedures, dates, scales, or the cutoff points of scales may result in varied prevalence rates (9), such as 12% in Greece (*N* = 1,104) (10) and nearly half in France (*N* = 619) (11). An online survey conducted among 11,787 Chinese college students in February 2020 showed that the prevalence rates of depressive [the Patient Health Questionnaire—nine-item (PHQ-9) ≥ 5] and anxiety [the seven-item Generalized Anxiety Disorder Scale (GAD-7) ≥ 5] symptoms were 25.9 and 17.8%, respectively (12). This study also found that traveling to or living in the outbreak “hotspot” area was associated with a higher risk of depressive and anxiety symptoms (12). Another survey from Guangdong Province in China found that the rate of depressive symptoms among college students was 7% (*N* = 361,969, PHQ-9 ≥ 10) (13).

Some studies have focused on the mental health status of medical students during epidemic outbreaks. Previous studies reported that medical students had increased anxious feelings after the severe acute respiratory syndrome (SARS) (14) and the Middle East respiratory syndrome coronavirus (MERS) outbreaks (15). Furthermore, varied prevalence rates of poor mental health status were reported in different areas after the COVID-19 outbreak. In February 2020, 25.3% of Chinese medical students (*N* = 933) were reported to have depressive symptoms (PHQ-9 ≥ 5) and 17.1% of them were with anxiety symptoms (GAD-7 ≥ 5) (16). A small-scale survey (*N* = 217) conducted in a Chinese university in February 2020 found that 37% of medical students had a serious mental illness [the Kessler 6 Psychological Distress Scale (K6) ≥ 12] (17). A comparative study found that 1,442 Chinese health professional students (including 764 medical students) experienced increased distress (K6 ≥ 5, 26.6%) and acute stress in February 2020 compared with that in the pre-pandemic in October 2019 (18). In addition, nearly half of the medical students in the United Arab Emirates had mild to severe anxiety symptoms (*N* = 1,485, GAD-7 ≥ 5) in March 2020 (19). During the initial period of the COVID-19 pandemic, 49.9% of 425 Bangladeshi medical students reported anxiety and 69.9% of them were with depressive symptoms through an online survey using the Hospital Anxiety and Depression Scale (20). A survey conducted in the United States (*N* = 741) also showed that COVID-19 disrupted medical education and clinical training and that medical students experienced moderate stress and anxiety, which was measured using one Likert item (2). In Pakistan, approximately one in five final-year medical students (*N* = 2,661) felt bored or nervous about the closure of their institutes because of the COVID-19 pandemic in June 2020 (21).

All of these studies among medical students did not explore the association between the severity of viral exposure and mental health. Additionally, mental health during a crisis is often related to social support (22). The government enforced “physical distancing” aimed at infection control, such as the transition from classroom learning to virtual learning, which may result in personal isolation (23). However, previous research has poorly studied the possible effect of social support on medical students' mental health during epidemic outbreaks. Studies during the COVID-19 pandemic showed that low-level social support was associated with a higher risk of mild to severe depressive or anxiety symptoms among Chinese college students (24), Chinese adolescents (25), and British pregnant women (26). More perceived social support was associated with a decreased risk of sleep disturbance and suicidal ideation among the Taiwanese population (27).

Thus, this study aimed to explore whether personal virus exposure, regional epidemic condition, and social support were associated with medical students' depressive and anxiety symptoms during the period of the fast-spreading COVID-19 outbreak in China. It would inform medical educators and health policymakers to take measures in order to make young health workforce or students mentally well-prepared for public emergencies.

## Method

This cross-sectional survey enrolled respondents between February 11, 2020 and February 18, 2020. The retrospective design collected information on the period from the fast outbreak at the end of January 2020 to the stable epidemic in the middle of February 2020. After the COVID-19 outbreak in China at the end of 2019, more than 30,000 health workers from other provinces were deployed to the frontlines to fight against the virus in Hubei Province (28). Due to high hospital-associated transmission risks (29), as of February 14, 2020, 1,716 health workers were diagnosed with COVID-19 because of hospital-acquired infections, and eight of them died (30).

We distributed online questionnaires through messaging and social media apps (WeChat and QQ) to medical school staff and students. Students' participation was anonymous and voluntary. They completed the questionnaires on the Wenjuanxing survey platform (https://www.wjx.cn/). All subjects were informed of an introduction to the study and provided online informed consent before starting the survey. They did not receive any reward.

Epidemic condition data were from the official daily briefings of the Health Commission of China or provincial health commissions (31).

The Institutional Review Board of Beijing HuiLongGuan Hospital approved this study.

### Participants

Medical students were defined as full-time undergraduate or graduate students majoring in clinical medicine. According to the medical practice policy in China, only those studying clinical medicine could be qualified to register to practice (modern medicine) in the future. After a 5-year undergraduate medical education program, medical students can schedule their National Medical Practitioner Examination the following year. After they pass the exam, acquire practice licenses, and complete 2 years of standardized residency training programs, they can finally seek registration to practice medicine.

We included medical students who were 18–35 years old and who finished all the questionnaires. Then, we excluded those who were living abroad or completed the questionnaires in <3 min.

### Measurement

Possible personal COVID-19 exposure was identified if the person was diagnosed with COVID-19, if the person's family members or close contacts were diagnosed with COVID-19, or if the person was under involuntary isolated care or observation.

The provincial epidemic condition was converted from the cumulative confirmed COVID-19 cases in the respondent's present residential province on the day before the answering day. It was categorized into four levels: “1–100,” “100–499,” “500–999,” and “1,000 and above.”

Depressive symptoms within the past 14 days were screened using the nine-item PHQ-9, with each of the nine DSM-IV criteria scored on a scale from 0 “not at all” to 3 “nearly every day” (32). The score ranges for symptom severity are 0–4 for minimal, 5–9 for mild, 10–14 for moderate, and 15–27 for severe (32). The scale was validated in Chinese university students with a clinically significant cutoff point of 10 (33). The majority of studies among medical students or students' mental health during the COVID-19 pandemic only presented results on mild to severe depressive symptoms using five as the cutoff point (3, 9, 12, 16, 34). For a better comparison with previous research and policy attention for subclinical symptoms, we used two cutoff points, namely a PHQ score ≥5 for mild to severe depressive symptoms and a PHQ score ≥10 for moderate to severe depressive symptoms. The Cronbach's α in the present study was 0.87.

Anxiety symptoms within 14 days were screened using the seven-item GAD-7, with items rated from 0 “not at all” to 3 “nearly every day” (35). The score ranges for symptom severity are 0–4 for minimal, 5–9 for mild, 10–14 for moderate, and 15–21 for severe. The clinical cutoff point at 10 was validated in China (36). Some studies on college students during the COVID-19 pandemic used five as a cutoff point for mild to severe anxiety symptoms (12, 16, 25). In our study, the result of the GAD score ≥5 for mild to severe anxiety symptoms and the GAD score ≥10 for moderate to severe anxiety symptoms were presented. The Cronbach's α in this study was 0.92.

Social support was assessed using the Social Support Rating Scale (SSRS), which measured subjective social support, objective social support, and support utility (37). The total score was categorized into three levels (“low,” “medium,” and “high”) using terciles as cutoff points because its distribution was skewed (38). The Cronbach's α in the present study was 0.95.

Recent emotions were collected using a multiple-choice question: “Please choose one or more words to describe your emotions toward the COVID-19 outbreak within the past seven days.” The seven emotion words were: “terrified,” “pessimistic,” “numb (detached),” “nervous,” “helpless,” “calm,” and “angry.”

Demographics included age, gender, years of training, and residence area (rural/urban). The 3 years of training subgroups were: undergraduates (years 1–3), undergraduates (years 4–5), and graduate students. In the Chinese 5-year medical curriculum, the preclinical phase included 3 or more years of education on medical concepts and science. Students usually start clinical education or internship at teaching hospitals once a week during their 4th year. In the 5th year, they engaged in full-time clinical rotations. Medical graduate students should complete residency training programs during their graduate education.

### Power

We used a sample size calculation for the logistic regression (39). According to previous research on the prevalence of depressive symptoms among medical students or college students (3, 9), we set *P*_*x*__= 0_ = 0.27, *P*_*x*__= 1_ = 0.40. Then, we assumed a detectable odds ratio (OR) of 1.2, α = 0.05, power = 0.8. Thus, the sample size would be 4,890.

### Statistical Analysis

The chi-squared test and Mann–Whitney rank-sum test examined differences in the rates of symptoms or emotions by the four ordered provincial epidemic conditions. Multivariable logistic regression tested the associations of COVID-19 exposure factors and social supports with depressive symptoms or anxiety symptoms, controlling for age, gender, years of training, and residence area. We presented the regression results for mild to severe depressive symptoms, moderate to severe depressive symptoms, mild to severe anxiety symptoms, and moderate to severe anxiety symptoms, respectively.

The software for statistical analysis was Stata 15.0 for Windows (StataCorp, College Station, TX, USA). Statistical significance was set at *P* < 0.05.

## Results

### Demographics

In total, 5,982 medical students participated the survey and finished all questions. The median age was 22 years [interquartile range (IQR) = 3, range = 18–35, mean = 21.7, standard deviation (SD) = 2.5], and 60.0% were female. The students came from all provincial regions of China except for Macau (Table 1). The majority (69.3%) were first to 3rd year undergraduates, approximately one in five were 4 and 5th year undergraduates, and the other were graduate students. Among them, 63.0% lived in rural areas.

**Table 1:** Demographics, epidemiological characteristics, and depressive or anxiety symptoms among Chinese medical students during the 2019 coronavirus disease (COVID-19) outbreak (*N* = 5,982).

**Variables**	***n***	**%**	**Depressive symptoms^a^**				**Anxiety symptoms^b^**			
			**Minimal (%)**	**Mild (%)**	**Moderate (%)**	**Severe (%)**	**Minimal (%)**	**Mild (%)**	**Moderate (%)**	**Severe (%)**
Age (years) median, IQR	22	3								
Total	5,982	100	64.8	25.3	6.6	3.2	77.2	18.6	2.3	1.8
Possible COVID-19 exposure										
No	5,433	90.8	65.6	24.7	6.6	3.1	78.0	17.9	2.3	1.8
Yes	549	9.2	56.8	31.3	7.3	4.6	69.6	25.7	2.4	2.4
Provincial epidemic condition^c^										
<100	389	6.5	69.9	19.5	6.4	4.1	79.9	15.4	2.6	2.1
100–499	4,894	81.8	65.0	25.3	6.5	3.2	77.5	18.3	2.4	1.8
500–999	305	5.1	59.3	31.8	6.9	2.0	71.8	24.3	2.3	1.6
1,000 and above	394	6.6	62.2	26.1	7.9	3.8	75.1	20.8	1.5	2.5
Social support^d^										
High	1,852	31.0	76.6	19.3	3.0	1.1	85.6	12.3	1.3	0.8
Medium	2,079	34.8	67.8	24.3	5.9	2.0	78.4	18.2	1.9	1.5
Low	2,051	34.3	51.2	31.8	10.6	6.4	68.5	24.7	3.7	3.1
Gender										
Male	2,391	40.0	69.1	22.6	5.7	2.6	80.0	16.4	2.0	1.6
Female	3,591	60.0	62.0	27.1	7.3	3.6	75.4	20.0	2.6	2.0
Year of training										
Undergraduates, years 1–3	4,146	69.3	64.2	25.9	6.7	3.3	77.2	18.9	2.1	1.9
Undergraduates, years 4–5	1,356	22.7	68.0	23.0	5.8	3.2	79.9	15.6	2.8	1.7
Graduate students	480	8.0	61.7	27.1	8.5	2.7	70.4	24.8	2.7	2.1
Residence										
Urban	2,213	37.0	65.3	24.4	6.9	3.5	78.2	17.5	2.6	1.7
Rural	3,769	63.0	64.6	25.9	6.5	3.0	76.7	19.2	2.2	1.9

*IQR, interquartile range*.

a *Categories for increasing depressive symptoms, which were measured using the Patient Health Questionnaire: 0–4 for minimal, 5–9 for mild, 10–14 for moderate, and 15–27 for severe*.

b *Categories for increasing anxiety symptoms, which were measured using the Generalized Anxiety Disorder Scale: 0–4 for minimal, 5–9 for mild, 10–14 for moderate, and 15–21 for severe*.

c *Provincial confirmed COVID-19 cases 1 day prior to the answering day. Data were from health commissions*.

d *The levels of social support were converted from the total scores for Social Support Rating Scales with terciles as cut points*.

*Results statistically significant at p < 0.05 level are in bold*.

### Epidemiological or Clinical Characteristics

The median scores for PHQ-9 was 3 (IQR = 6, range = 0–27) and that for GAD-7 was 1 (IQR = 4, range = 0–21). Approximately one-third (35.2%) of the respondents had mild to severe depressive symptoms, and one in 10 (9.8%) had moderate to severe depressive symptoms (Table 1). More than one-fifth of patients (22.8%) had mild to severe anxiety symptoms. Only 4.2% of patients had moderate to severe anxiety symptoms. The Cohen's *d* of both the PHQ score and the GAD score between with and without personal exposure was 0.18.

Nearly one in ten (9.2%) respondents were at possible risk due to personal exposure to COVID-19 (Table 1), and 26 of them reported that they or their family members were infected. Those with COVID-19 exposure had an elevated severity of depressive [$\chi_{\left(3\right)}^{2}$ = 18.35, *P* < 0.001] and anxiety [$\chi_{\left(3\right)}^{2}$ = 21.89, *P* < 0.001] symptoms than those without COVID-19 exposure.

Two in five (40.7%) respondents living in provinces with 500–999 confirmed cases had mild to severe depression, which was higher than that of others [1,000 and above confirmed cases, 37.8%; 100–499 cases, 35.0%; 1–100 cases, 30.1%; $\chi_{\left(9\right)}^{2}$ = 17.67, *P* = 0.039] (Table 1).

The highest rate of mild to severe anxiety symptoms was also among respondents in provinces with 500–999 confirmed cases (28.2%), followed by those in provinces with over 1,000 provincial cases (24.9%), 100–499 cases (22.5%), and 1–100 cases (20.0%). However, the association between provincial epidemic condition and anxiety symptoms was insignificant [$\chi_{\left(9\right)}^{2}$ = 13, *P* = 0.162].

The median score for social support was 36 (IQR = 9, range = 11–60). Increased social support was associated with a decreased severity of depressive [$\chi_{\left(6\right)}^{2}$ = 350.62, *P* < 0.001] and anxiety [$\chi_{\left(3\right)}^{2}$ = 174.83, *P* < 0.001] symptoms.

### Emotions Within 7 Days

Approximately three-fourths (72.5%) of the respondents stated that they felt calm about the COVID-19 outbreak within 7 days, and nearly half (47.7%) reported that they felt nervous. Approximately one in five reported that they felt angry (20.2%) or terrified (18.3%). Helpless, numb, and pessimistic emotions were experienced by 12.6, 10.7, and 8.1% of the respondents, respectively.

Respondents living in a severe provincial epidemic condition reported more numbness (detached feelings) about the outbreak (*z* = 2.71, *P* = 0.007): <100 cumulative COVID-19 cases, 14.7%, 100–499, 11.1%, 500–999, 10.6%, and 1,000 and above, 8.2%. Other emotions did not vary significantly between the different provincial epidemic conditions (all *P* > 0.05).

### Related Factors for Depressive Symptoms

Logistic regression (Table 2, model 1) showed that the risk factors for mild to severe depressive symptoms were low-level social support [adjusted odds ratio (AOR) = 3.19, 95% CI = 2.77–3.67] or medium-level social support (AOR = 1.53, 95% CI = 1.33–1.76), female gender (AOR = 1.48, 95% CI = 1.32–1.65), 500–999 provincial confirmed cases (AOR = 1.50, 95% CI = 1.08–2.08), and COVID-19 exposure (AOR = 1.33, 95% CI = 1.11–1.61). 4 or 5th year undergraduates reported fewer depressive symptoms (AOR = 0.81, 95% CI = 0.69–0.95) compared with other undergraduates. Residence areas (urban/rural) were not associated with depressive symptoms. No interaction effect was observed in the model.

**Table 2:** Logistic regression on depressive symptoms among Chinese medical students during the 2019 coronavirus disease (COVID-19) outbreak (*N* = 5,982).

**Variables**	**Model 1: mild to severe depressive symptoms^a^**				**Model 2: moderate to severe depressive symptoms^b^**			
	**AOR**	**95% CI**		***P***	**AOR**	**95% CI**		***P***
		**LL**	**UL**			**LL**	**UL**	
Possible COVID-19 exposure								
No	1.00				1.00			
Yes	**1.33**	**1.11**	**1.61**	**0.003**	1.12	0.84	1.49	0.434
Provincial epidemic condition^c^								
<100	1.00							
100–499	1.20	0.95	1.51	0.129	0.85	0.60	1.21	0.376
500–999	**1.50**	**1.08**	**2.08**	**0.015**	0.75	0.44	1.26	0.278
1,000 and above	1.26	0.92	1.72	0.142	0.98	0.62	1.56	0.942
Social support^d^								
High	1.00				1.00			
Medium	**1.53**	**1.33**	**1.76**	** <0.001**	**1.98**	**1.50**	**2.62**	** <0.001**
Low	**3.19**	**2.77**	**3.67**	** <0.001**	**4.89**	**3.78**	**6.33**	** <0.001**
Age (years)	1.00	0.97	1.04	0.871	1	0.95	1.05	0.943
Gender								
Male	1.00				1.00			
Female	**1.48**	**1.32**	**1.65**	** <0.001**	**1.47**	**1.22**	**1.77**	** <0.001**
Grade								
Undergraduates, years 1–3	1.00				1.00			
Undergraduates, years 4–5	**0.81**	**0.69**	**0.96**	**0.014**	0.89	0.68	1.16	0.377
Graduate students	1.00	0.76	1.38	0.869	1.12	0.71	1.78	0.626
Residence								
Urban	1				1			
Rural	1.07	0.95	1.20	0.252	0.95	0.79	1.14	0.581

*AOR, adjusted odds ratio; CI, confidence interval; LL, low level; UL, upper level*.

a *Scores for the Patient Health Questionnaire ≥ 5*.

b *Scores for the Patient Health Questionnaire ≥ 10*.

c *Provincial confirmed COVID-19 cases 1 day prior to the answering day. Data were from health commissions*.

d *The levels of social support were converted from the total scores for Social Support Rating Scales with terciles as cut points*.

*Results statistically significant at p < 0.05 level are in bold*.

Moreover, when the analysis was repeated using moderate to severe depressive symptoms as the dependent variable, the odds ratio of COVID-19 exposure or provincial epidemic condition was insignificant (*P* > 0.05; Table 2, model 2). The effect of low-level social support (AOR = 4.89, 95% CI = 3.78–6.33), medium-level social support (AOR = 1.98, 95% CI = 1.50–2.62), and female gender (AOR = 1.47, 95% CI = 1.22–1.77) remained significant.

### Related Factors for Anxiety Symptoms

Female gender (AOR = 1.36, 95% CI = 1.20–1.55), living in rural areas (AOR = 1.14, 95% CI = 1.00–1.30), possible COVID-19 exposure (AOR = 1.40, 95% CI = 1.15–1.71), and medium-level (AOR = 1.62, 95% CI = 1.37–1.92) or low-level (AOR = 2.78, 95% CI = 2.37–3.27) social support were associated with an elevated risk of mild to severe anxiety symptoms (Table 3, model 1). Fourth or Fifth year undergraduates (AOR = 0.79, 95% CI = 0.65–0.95) had lower anxiety symptom rates than 1st to 3rd year undergraduates. No significant relationship was found between the provincial epidemic condition and anxiety symptoms (*P* > 0.05). However, the interaction effect was not statistically significant.

**Table 3:** Logistic regression on anxiety symptoms among Chinese medical students during the 2019 coronavirus disease (COVID-19) outbreak (*N* = 5,982).

**Variables**	**Model 1: mild to severe anxiety symptoms^a^**				**Model 2: moderate to severe anxiety symptoms^b^**			
	**AOR**	**95% CI**		***P***	**AOR**	**95% CI**		***P***
		**LL**	**UL**			**LL**	**UL**	
Possible COVID-19 exposure								
No	1.00				1.00			
Yes	**1.40**	**1.15**	**1.71**	**0.001**	1.08	0.71	1.66	0.715
Provincial epidemic condition^c^								
<100	1.00							
100–499	1.10	0.85	1.43	0.469	0.82	0.49	1.35	0.427
500–999	1.40	0.98	2.02	0.065	0.77	0.36	1.64	0.498
1,000 and above	1.11	0.78	1.57	0.559	0.77	0.38	1.55	0.456
Social support^d^								
High	1.00				1.00			
Medium	**1.62**	**1.37**	**1.92**	** <0.001**	**1.64**	**1.10**	**2.44**	**0.015**
Low	**2.78**	**2.37**	**3.27**	** <0.001**	**3.45**	**2.40**	**4.95**	** <0.001**
Age (years)	1.02	0.99	1.06	0.241	1.04	0.96	1.12	0.383
Gender								
Male	1.00				1.00			
Female	**1.36**	**1.20**	**1.55**	** <0.001**	**1.35**	**1.03**	**1.76**	**0.031**
Grade								
Undergraduates, years 1–3	1.00				1.00			
Undergraduates, years 4–5	**0.79**	**0.65**	**0.95**	**0.011**	1.03	0.71	1.49	0.895
Graduate students	1.16	0.85	1.60	0.351	0.98	0.50	1.94	0.959
Residence								
Urban	1				1			
Rural	**1.14**	**1.00**	**1.30**	**0.043**	0.99	0.75	1.29	0.915

*AOR, adjusted odds ratio; CI, confidence interval; LL, low level; UL, upper level*.

a *Scores for the Generalized Anxiety Disorder Scale ≥ 5*.

b *Scores for the Generalized Anxiety Disorder Scale ≥ 10*.

c *Provincial confirmed COVID-19 cases 1 day prior to the answering day. Data were from health commissions*.

d *The levels of social support were converted from the total scores for Social Support Rating Scales with terciles as cut points*.

*Results statistically significant at p < 0.05 level are in bold*.

Furthermore, possible COVID-19 exposure and the provincial epidemic condition were not associated with an elevated risk of moderate to severe anxiety symptoms (all *P* > 0.05; Table 3, model 2). Low-level social support (AOR = 3.45, 95% CI = 2.40–4.95), medium-level social support (AOR = 1.64, 95% CI = 1.10–2.44), and female gender (AOR = 1.35, 95% CI = 1.03–1.76) increased the risk of these symptoms.

## Discussion

This large-scale survey demonstrated that most of the medical students had good mental status and calm feelings. Furthermore, mild depressive symptoms and anxiety symptoms were relatively common. Possible exposure to COVID-19 and provincial epidemic spread were associated with a slightly increased risk of poor mental health, while low social support was a more relevant factor.

The prevalence rates of mild to severe depressive (35%) and anxiety (23%) symptoms in our study were higher than those in another survey of Chinese medical students (25.3% for depressive symptoms and 17.1% for anxiety symptoms) that was conducted during a similar period using the same scales and cutoff points (16). Both studies used convenience sampling, but our sample size was much larger. The rates of depressive and anxiety symptoms in the medical students in our study were higher than those reported in a Chinese college student survey (25.9% for depressive symptoms and 17.8% for anxiety symptoms) in February 2020, which used the same cutoff points for the same scales (12). In addition, the rates in our study were also higher than the rates among Chinese medical students on normal days: 29% for depressive symptoms and 21% for anxiety symptoms (40). The small number of elevated mental problems may have occurred because the COVID-19 outbreak happened during the Spring Festival, the most important family gathering for Chinese people. Most students were at home rather than at universities or medical settings, meaning that they had more family support and lower occupational infection risk. The low prevalence rate of anxiety may be related to the decisive governance of the Chinese central government, social mobilization, mass official health education, and timely news updates from official media (41).

COVID-19 exposure increased the likelihood of mild to severe depressive or anxiety symptoms more than the provincial epidemic condition. Being an infected person or with family caregivers fighting against a communicable disease means directly facing death, loss, care-seeking, and even financial issues (42). These life events may be more related to poor mental health than provincial epidemic conditions. When the number of provincial confirmed cases of COVID-19 was between 500 and 1,000, the medical students expressed more depressive symptoms. The initial rapid COVID-19 outbreak required people in their respective regions to quickly adjust their minds and lifestyle and adapt to the “new normal.” Therefore, we found that the association between the depression rate and the severity of the provincial epidemic condition was not linear but like an inverted “U” even after being adjusted for personal virus exposure and other factors. Furthermore, it is surprising that the relationship between provincial epidemic conditions and anxiety symptoms was insignificant. Another study among Chinese college students showed that confirmed COVID-19 cases in the current residence area were not associated with depressive (PHQ ≥ 5) or anxiety (GAD ≥ 5) symptoms (12). However, their study only presented crude odds ratios rather than ratios adjusted for demographics and social support. Then, it is important to note that, when using different cutoff points of scales, the epidemic-related factors were not related to moderate to severe depressive or anxiety symptoms. In other words, the impact of the epidemic may only increase mild mental problems in this sample. The elevated symptom severity might be a normal sadness and disturbance response to an unprecedented health crisis (43). Major depressive disorder or general anxiety disorder may be a result of an interaction between genetic factors and lifetime environmental conditions rather than COVID-related factors. Another study found that, compared to people without mental disorders, those with the greatest mental disorder burden had decreased depressive symptoms and worries during the COVID-19 pandemic (43). Pandemic exposure may only be a relatively small stressor for those with poor mental health pre-pandemic. In addition, students living in difficult provincial epidemic conditions expressed more numbness. This feeling seems to be one of the acute stress symptoms caused by the epidemic.

Compared with the epidemic-related factors, factors associated with poor mental health on normal days showed more potent effects. The risk of depressive or anxiety symptoms for medical students with low-level social support was approximately three times more than those with high-level social support. This result is similar to previous findings among medical students under normal conditions (44). In addition, the association between social support and poor mental health status is consistent with studies conducted among other populations during the COVID-19 pandemic (24–26). Poor mental health status among female medical students was also reported by other studies after COVID-19 (16, 18, 19) or the MERS outbreak (15), while this gender difference was not significant on normal days (40). The lower risk of depression among fourth and fifth year students is consistent with a previous meta-analysis (5). Comparatively, preclinical undergraduates had more curricula to complete and less knowledge about medicine. Some graduate students needed to be engaged and immersed in the clinical environment because of healthcare workforce shortage, and some of them also had to conduct research tasks in their labs.

The findings of this study highlight the importance of improving the social support of medical students. Physical distancing, as a method to avoid virus transmission, also reduces social activities that keep people mentally healthy. Universities and colleges can provide online psychological support, hotline services, or online courses about psychological preparations for disasters or public crises. When students return to teaching hospitals, they should ensure students' occupational safety, provide supervision, and fully train them on COVID-19. In addition, it is necessary to screen the epidemiological history and mental health status of some high-risk medical students when they return to medical schools or teaching hospitals. Female students, those with low social support, or those who have experienced negative life events associated with the COVID-19 pandemic should receive assistance through possible referrals when the results of screens are positive. Considering our finding that the depressive and anxiety symptoms were generally mild, self-help methods and mental health education programs would also be helpful. Some students had negative feelings, such as anger and hopelessness; thus, emotional expression or management may be important. Furthermore, resilience training (45) can be integrated into medical education to help students become more competent in future health emergency challenges. More infection treatment experience or training may also increase their self-efficacy and intention to provide services or care for patients infected with a newly discovered virus (46).

### Strengths and Limitations

The most significant strength of this study is that, to our knowledge, it is the largest survey on the mental health of medical students at a very unique and important window of time during an emerging communicable disease. Using data from a large country with a strong “lockdown” policy made it possible to explore the association between regional epidemic severity and mental status in a “natural trial.” Then, we differentiated personal virus exposure as a “small” environment and provincial epidemic condition as a “large” environment to test their independent impacts. Furthermore, matching personal data with official provincial epidemic data also minimized reporting bias. Finally, this study not only identified the negative mental effects of the outbreak (e.g., depression and anxiety) but also demonstrated the students' positive feelings, such as calmness, providing a complete picture of their experience.

However, there are several limitations. Firstly, the recruitment of participants was conducted through convenience sampling. We could not give a response rate or weigh this sample to increase the representativeness because statistics on national medical students (only majoring in clinical medicine) were not available. The total number of students in medical schools, including students majoring in nursing, pharmacy, or basic medical science, was ~3 million (47). In addition, the infected or virus-exposed medical students may not want to participate in this survey; therefore, the generalization of the rates of depression and anxiety was limited. Secondly, this study did not collect the locations of the medical schools. While most of the students were at home because of the winter vacation, the epidemic condition where their colleges or universities were located could also impact them, for example, through postponement of the return to medical school or a high risk of virus exposure in teaching hospitals. Thirdly, we did not collect information about their socio-economic status and parents' education level, which may have confounded the results. Finally, an online survey cannot validate the respondents' identities, and self-reports may accompany information bias despite an anonymous data collection process.

In conclusion, nearly one in three Chinese medical students had mild to severe depressive symptoms during the COVID-19 outbreak. Over one-fifth of the patients had mild to severe anxiety symptoms. The risk of depressive or anxiety symptoms for medical students with low social support was higher than those with medium- or high-level social support. COVID-19 exposure increased the risk of depressive and anxiety symptoms. Initial rapid increase in provincial confirmed COVID-19 cases was positively related to mild to severe depressive symptoms. However, COVID-19 exposure and the severity of provincial epidemic conditions were not associated with moderate to severe depressive or anxiety symptoms. Correlates for poor mental status on normal days, such as low social support, female gender, and classification of pre-clerkship or graduates, should be highlighted. Some high-risk medical students need more social support.

## Data Availability Statement

The datasets presented in this article are not readily available because of ongoing analyses. Requests to access the datasets should be directed to chenjx1110@163.com.

## Ethics Statement

The studies involving human participants were reviewed and approved by Institutional Review Board of Beijing HuiLongGuan Hospital. All subjects were informed of an introduction to the study and provided online informed consent before starting the survey.

## Author Contributions

JC and YT conceptualized and designed the study. XY, LG, SZ, MQ, and LZ acquired the data. YY analyzed and interpreted the data. YY and JC drafted the manuscript. YT, XY, LG, SZ, MQ, and LZ critically revised the manuscript for important intellectual content. All authors gave final approval of the version and agreed to be accountable for all aspects of the work in ensuring questions related to the accuracy or integrity.

## Conflict of Interest

The authors declare that the research was conducted in the absence of any commercial or financial relationships that could be construed as a potential conflict of interest.
